# Defining and identifying satellite cell-opathies within muscular dystrophies and myopathies

**DOI:** 10.1016/j.yexcr.2021.112906

**Published:** 2022-02-01

**Authors:** Massimo Ganassi, Francesco Muntoni, Peter S. Zammit

**Affiliations:** a Randall Centre for Cell and Molecular Biophysics, King's College London, London, SE1 1UL, UK; b Dubowitz Neuromuscular Centre, UCL Great Ormond Street Institute of Child Health, 30 Guilford Street, London, WC1N 1EH, United Kingdom; c NIHR Great Ormond Street Hospital Biomedical Research Centre, UCL Great Ormond Street Institute of Child Health, 30 Guilford Street, London, WC1N 1EH, United Kingdom

**Keywords:** Muscle stem cell, Satellite cell, Satellite Cell-opathy, Skeletal muscle, Congenital myopathy, Muscular dystrophy, PAX7, Myopathogene, Carey-Fineman-Ziter Syndrome, CFZS, Creatine Kinase, CK, Duchenne muscular dystrophy, DMD, Dystrophin-Associated Protein Complex, DAPC, Facioscapulohumeral muscular dystrophy, FSHD, Gene Ontologies Satellite Cells, GOSC, Glycogen Storage Disease II, GSD2, Limb-Girdle Muscular Dystrophy Recessive 1, LGMDR1, Myopathy, Areflexia, Respiratory Distress, And Dysphagia, Early-Onset, EMARDD, Myopathy, Congenital, With Diaphragmatic Defects, Respiratory Insufficiency, And Dysmorphic Facies, MYODRIF, Myopathy, Congenital, With Fiber-Type Disproportion, CFTD, Muscular Dystrophy, Congenital, Lmna-Related, MDCL, Muscular Dystrophy, Congenital Merosin-Deficient, 1a MDC1A, Progressive Congenital Myopathy with Scoliosis, MYOSCO, Rigid Spine Muscular Dystrophy 1, RSMD1, Ulrich congenital muscular dystrophy, UCMD

## Abstract

Muscular dystrophies and congenital myopathies arise from specific genetic mutations causing skeletal muscle weakness that reduces quality of life. Muscle health relies on resident muscle stem cells called satellite cells, which enable life-course muscle growth, maintenance, repair and regeneration. Such tuned plasticity gradually diminishes in muscle diseases, suggesting compromised satellite cell function. A central issue however, is whether the pathogenic mutation perturbs satellite cell function directly and/or indirectly via an increasingly hostile microenvironment as disease progresses. Here, we explore the effects on satellite cell function of pathogenic mutations in genes (myopathogenes) that associate with muscle disorders, to evaluate clinical and muscle pathological hallmarks that define dysfunctional satellite cells. We deploy transcriptomic analysis and comparison between muscular dystrophies and myopathies to determine the contribution of satellite cell dysfunction using literature, expression dynamics of myopathogenes and their response to the satellite cell regulator PAX7. Our multimodal approach extends current pathological classifications to define Satellite Cell-opathies: muscle disorders in which satellite cell dysfunction contributes to pathology. Primary Satellite Cell-opathies are conditions where mutations in a myopathogene directly affect satellite cell function, such as in Progressive Congenital Myopathy with Scoliosis (MYOSCO) and Carey-Fineman-Ziter Syndrome (CFZS). Primary satellite cell-opathies are generally characterised as being congenital with general hypotonia, and specific involvement of respiratory, trunk and facial muscles, although serum CK levels are usually within the normal range. Secondary Satellite Cell-opathies have mutations in myopathogenes that affect both satellite cells and muscle fibres. Such classification aids diagnosis and predicting probable disease course, as well as informing on treatment and therapeutic development.

## Highlights

• Muscle homeostasis, growth and repair are impaired in muscular dystrophies and myopathies, exposing satellite cell dysfunction.
• Our multimodal approach identifies ‘myopathogenes’ in muscle disorders that could directly alter satellite cell function.
• In ‘Primary Satellite Cell-opathies’ the myopathogene directly affects satellite cell function.
• In ‘Secondary Satellite Cell-opathies’ the myopathogene affects both the function of satellite cells and muscle fibres.

## 1 Introduction

Most tissues and organs in human are capable of varying degrees of repair and regeneration, providing continuous and active adaptability to both endogenous and exogenous stimuli. Efficient renewal of systems also maintains homeostasis by restoring tissue functionality after intense use or trauma. Skeletal muscle accounts for ∼38% of total weight in men and ∼30% for women [1]. Beyond force generation, muscle is also involved in skeletal support, thermoregulation, and metabolism. Therefore, pathological conditions impinging on muscle maintenance and function severely impact the quality of life.

**Fig. 1:**
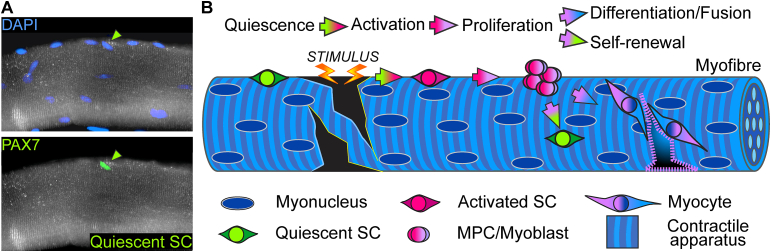
**Satellite cell myogenesis**. **A**. Representative image of a human quadriceps myofibre showing a quiescent satellite cell expressing PAX7 (green arrowheads; green nucleus, bottom) with myonuclei (blue) stained with DAPI. **B**. Satellite cells (SC) normally lay mitotically quiescent (green) between the basal lamina and plasmalemma of most myofibres. In response to stimuli (growth, trauma, disease), quiescent satellite cells are activated (red) and proliferate to generate a population of myoblasts (pink - lilac). A proportion of myoblasts undergo self-renewal to replenish the quiescent pool (green) on the myofibre, ensuring future stem cell function. Other myoblasts enter differentiation, becoming myocytes (lilac - blue) that fuse to contribute new myonuclei (blue) to pre-existing multinucleated muscle fibres or fuse together to form new myofibres.

The force-generating unit of skeletal muscle is the muscle fibre (myofibre), a syncytial cell formed from fusion of muscle progenitor cells during development and growth. Post mitotic myonuclei control the common cytoplasm packed with myofibrils composed of sarcomeres that generate force by shortening via interaction between actin and myosin filaments [[2], [3], [4], [5]]. Skeletal muscle is subject to persistent physical stress and thus relies on its ability to repair and locally regenerate. The resident population of stem cells for muscle fibres are named satellite cells, the key players in myofibre homeostasis, growth, hypertrophy, repair and regeneration [[6], [7], [8]]. Satellite cells reside on muscle fibres, beneath their basal lamina, a position that allows a prompt response [9,10] (Fig. 1A). Upon stimulus, satellite cells activate from their mitotically quiescent state, proliferate and generate a population of muscle progenitor cells called myoblasts, which then either differentiate and fuse into existing myofibres to supply new myonuclei for growth and repair, or fuse together to form nascent muscle fibres [11] (Fig. 1B). Satellite cell progeny are also able to self-renew to maintain the stem cell pool (Fig. 1B). Hence, satellite cells are essential for muscle growth, repair and regeneration, as demonstrated by genetic ablation experiments in mice [12].

Among the most common diseases detrimental to skeletal muscle are the muscular dystrophies and inherited myopathies, a heterogeneous group of genetic conditions characterised by muscle weakness (as defined in Refs. [[13], [14], [15]] and https://www.ninds.nih.gov/Disorders/All-Disorders/Myopathy-Information-Page). Myopathies present disorganised myofibre structure such as central nucleation or areas of myofibrillar disruption (minicores) as well as altered fibre-type proportions, often with congenital onset. Muscular dystrophies are characterised by repeated cycles of myofibre degeneration and regeneration, leading to gradual replacement of muscle tissue with fat, inflammatory infiltrates and fibrosis [16,17]. Notably, while histopathological features are usually distinct, poor regeneration capacity is generally common to both muscular dystrophies and myopathies.

Classification of muscular conditions depends on the genetic cause, pattern of Mendelian inheritance (X-linked/Autosomal; dominant/recessive) and muscle groups affected. Classification is also based on the age of onset; from congenital disorders evident at birth/during the first 6 months, to later-onset conditions, with some not even diagnosed until late adulthood, together with the rate of progression [14,18]. It is crucial to determine genetic aetiology in such conditions, since histopathological and clinical features of a muscular dystrophy or myopathy also depends on the expression profile and function of the mutated gene. Genetic diagnosis thus serves four important functions: 1) provides definitive classification in addition to clinical features, 2) helps clarify genotype-phenotype correlations, since the same clinical presentation can result from mutations in different genes, or mutations in the same gene lead to different pathologies, 3) facilitates better clinical management and 4) informs development of tailored therapies.

Currently, mutations in over 170 genes (hereafter termed myopathogenes) are linked to muscle disorders with genetic aetiology, comprising eight categories in the 2020 gene table for neuromuscular disorders [15] including dystrophies, myopathies, myotonic syndromes and ion-channel muscle diseases. Most mutations affect genes encoding proteins involved in maintenance of muscle architecture or contractility, such as in *NEBULIN* in Nemaline myopathy (NEM2; OMIM: 256030) [19]. However, some muscle diseases originate from genetic alterations impinging directly on satellite cell function and their ability to repair/regenerate myofibres, such as mutations in *PAX7* causing Progressive congenital myopathy with scoliosis (MYOSCO; OMIM: 618578).

Our aim is to better define the contribution of satellite cell dysfunction to the pathogenesis of inherited muscular dystrophies and myopathies. For such analysis, we selected the subset of 116 myopathogenes associated with four of the eight categories of muscle-specific conditions: namely muscular dystrophies, congenital muscular dystrophies, congenital myopathies and distal myopathies [15]. First, we collected all Gene Ontologies containing ‘satellite cell’ to create the term ‘Gene Ontologies Satellite Cells’ (GOSC), which together contained just 30 annotated genes. We then describe known muscular dystrophies/myopathies associated with mutation in any of these genes directly involved in satellite cell maintenance and function. We also discuss muscle disorders that should be included in this category. This leads to our classification of ‘Primary satellite cell-opathies’ for conditions primarily caused by perturbed satellite cell function. Mutations that affect both satellite cells and muscle fibres can then be classified as ‘Secondary satellite cell-opathies’. Finally, ‘Non-satellite cell-opathy neuromuscular disorders’ are where satellite cell function is not directly affected by the causative mutation. Next, to identify further potential satellite cell-opathies, we developed a strategy to evaluate expression and involvement of the selected 116 myopathogenes in early satellite cell function. This multimodal system involves transcriptomic analysis and comparisons of publicly available datasets, combined with consideration of whether expression of the myopathogene responds to the satellite cell-specific transcription factor PAX7. Where data is available, consideration of whether satellite cells are affected in the associated human disease and animal models can also be included.

## 2 Gene mutations that cause primary satellite cell-opathies

In healthy muscle, satellite cell activity is concomitant with maintenance of the stem cell pool to secure satellite cells for future need. After activation and proliferation, a proportion of satellite cell-derived myoblasts commit to self-renewal (Fig. 1B) [20,21]. This provides persistent repair/regeneration potential, allowing the muscle to adapt and repair through life. However, mutations altering expression or function of genes involved in satellite cell/myoblast activity would perturb muscle homeostasis and repair. Since regenerative myogenesis is often compromised in both myopathies and dystrophies, this implies defective satellite cell function.

**Fig. 2:**
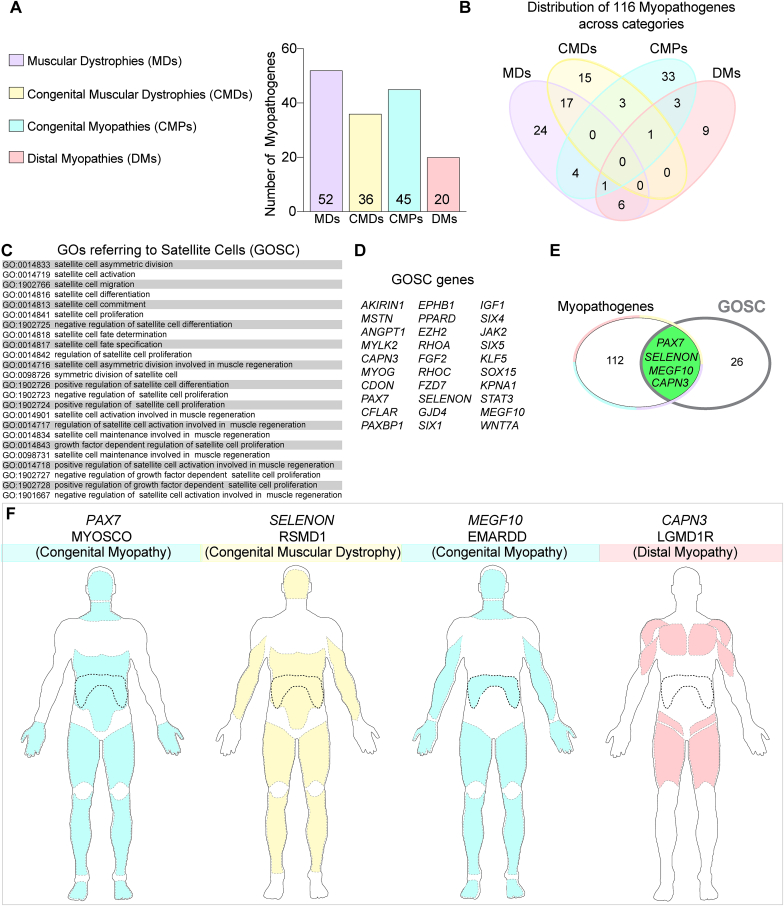
**Myopathogenes causing muscular dystrophies and myopathies and the subset within the Gene Ontology Satellite Cell term with their associated muscle disorders**. **A.** Distribution of the 116 myopathogenes [15] between human inherited muscular dystrophies (MDs), congenital muscular dystrophies (CMDs), congenital myopathies (CMPs) and distal myopathies (DMs). **B.** Venn diagram showing the number of myopathogenes shared between the selected muscle disease groupings. **C.** Gene Ontologies (GOs) containing ‘Satellite Cell’ comprising the term Gene Ontology Satellite Cell (GOSC). **D.** This novel GOSC contains 30 satellite cell-associated annotated genes. **E.** Venn diagram showing that four of the myopathogenes involved in satellite cell function in GOSC are associated with muscular dystrophy/myopathy when mutated: *PAX7*, *SELENON* (formerly *SEPN1*)*, MEGF10* and *CAPN3* (green overlap). **F.** Predominant muscle groups (dotted black line depicts diaphragm) affected by mutations in *PAX7* (MYOSCO), *SELENON* (RSMD1), *MEGF10* (EMARDD) and *CAPN3* (LGMD1R). Each disorder is colour coded as per A and B to indicate category.

To define the contribution of satellite cell dysfunction to muscular dystrophies and myopathies, we evaluated the 116 myopathogenes involved in muscular dystrophies, congenital muscular dystrophies, congenital myopathies and distal myopathies [15] (Fig. 2A and B). We first created the novel term ‘Gene Ontologies Satellite Cells (GOSC)’ by collating the current Gene Ontologies (GOs) that contain the word ‘satellite cell’ (Fig. 2C). GOSC comprises of 30 annotated genes (Fig. 2D). Of these 30 GOSC genes selected on the basis of involvement in satellite cell biology, only 4 are also included in the 116 myopathogenes, so known to cause muscular dystrophies/myopathies when mutated: these are *PAX7*, *MEGF10*, *SELENON* (formerly *SEPN1*) and *CAPN3* (Fig. 2E). Analysing the pathologies associated with mutations in these 4 genes at the clinical, cellular and molecular level, and focussing on the effects on satellite cell and muscle fibre function, provides a blueprint for defining ‘primary satellite cell-opathies’.

### 2.1 PAX7: progressive congenital Myopathy with scoliosis (MYOSCO)

Considering its central role in satellite cell specification and function, it is unsurprising that mutations in the transcription factor PAX7 cause a myopathy. The first evidence linking genetic alteration in human *PAX7* with a neuromuscular syndrome was reported in 2017 [22]. Patients display severe global neurodevelopmental delay, failure to thrive and axial hypotonia, suggestive of neuromuscular involvement. No visible signs of dystrophy were reported, such as alterations in muscle composition or fibrotic/adipose infiltrates, indicating minor effects on myofibre homeostasis, but an increase in regenerating myofibres was noted. A c.1403-2A > G autosomal recessive mutation was identified that maps to the intron splice site acceptor before *PAX7* exon 9 encoding for the PHT/OAR domain. With mRNA splicing possibly affected, the variant decreased *PAX7* isoform 3 transcripts, feasibly through non-sense mediated decay, but nuclear accumulation of PAX7 protein was reported in patient-derived myogenic cells [22].

**Fig. 3:**
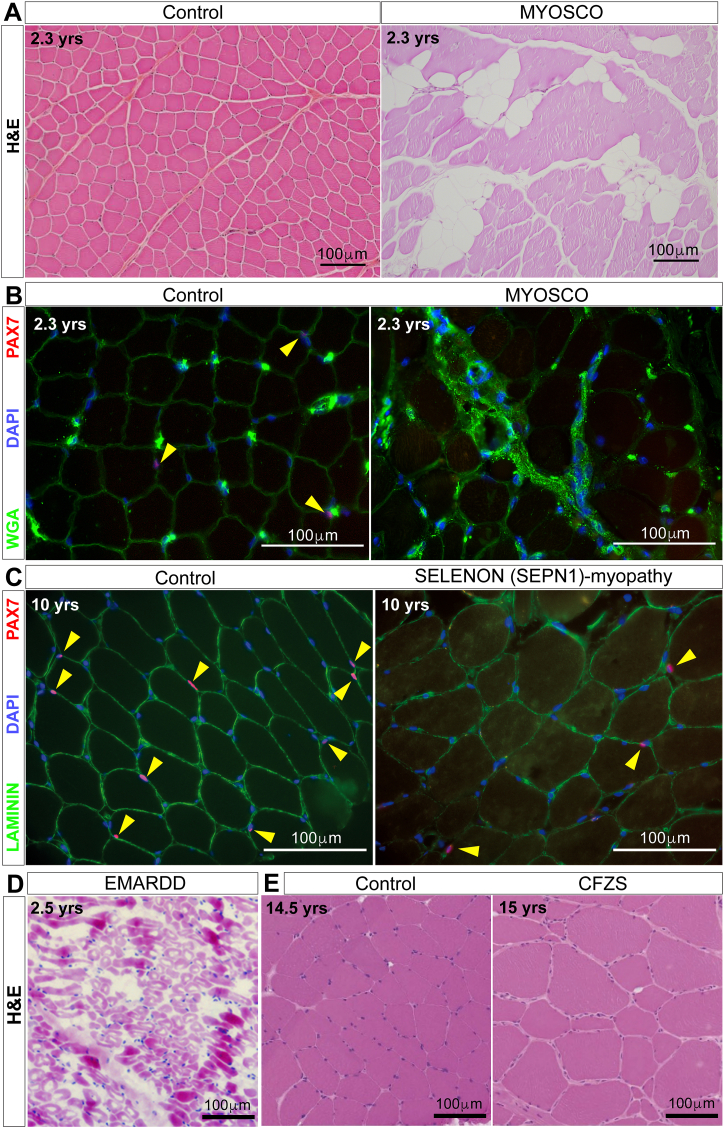
**Muscle pathology in MYOSCO, SELENON (SEPN1)-related, EMARDD and CFZS**. **A.** Representative Haematoxylin and Eosin (H&E) staining on MYOSCO quadriceps muscle biopsy exhibiting myopathology, notably areas of fat infiltration, compared to age-matched unaffected muscle (Control). **B.** Representative PAX7 immunolabelling demonstrating absence of satellite cells (PAX7-positive cells are red, indicated by yellow arrowheads) in MYOSCO compared to age-matched unaffected muscle (Control). Myofibres are delimited by Wheat germ agglutinin (WGA, green) and nuclei stained with DAPI (blue). **C.** Representative PAX7 immunolabelling showing reduction of satellite cells (PAX7-positive cells are red, indicated by yellow arrowheads) in SELENON (SEPN1)-myopathy compared to age-matched unaffected muscle (Control). Myofibres delimited by LAMININ (green) and nuclei identified with DAPI (blue). **D.** Representative H&E staining on an EMARDD muscle biopsy illustrating variation in myofibre size and fat/fibrotic infiltration. **E.** Representative H&E staining on CFZS muscle highlighting myofibre hypertrophy compared to age-matched unaffected muscle (Control) (adapted from Ref. [98]). The age of subjects at the time of muscle biopsy are reported, together with 100 μm scale bars.

More recently, four loss-of-function variants mapping within *PAX7* exons 1–3 have been linked to a myopathy with congenital onset and autosomal-recessive inheritance, referred to as progressive congenital myopathy with scoliosis (MYOSCO; OMIM: 618578) [23,24]. These patients also have generalised hypotonia and a growth deficit, aggravated by variable hypotrophy/atrophy affecting mainly muscles of the trunk, neck, leg, distal extremities and face (Fig. 2F). Concomitant weakness in diaphragm led to respiratory insufficiency. Strikingly, there was mild to no overt pathological features in muscle biopsies, apart from regions of fat/fibrotic infiltrations, minor atrophic patches and some areas of active regeneration (Fig. 3A). It is of note in general though that diagnostic biopsies are usually collected from readily accessible, large muscles, such as the vastus lateralis of the quadriceps, which may not always be representative of dystrophic changes in more affected muscles. However, in line with limited pathological signs, serum levels of Creatine Kinase (CK) are within the normal range [23] (Table 1).

Molecular and cellular analysis highlighted a strong decrease in *PAX7* expression, with complete absence of PAX7-positive cells, indicating a dramatic effect on satellite cells in MYOSCO patients (Fig. 3B). Expression of embryonic myosin heavy chain 3 (*MYH3*) identifying immature muscle fibres, indicated limited regenerative capacity. Indeed, patient-derived myoblasts could contribute to myonuclear accretion in myotubes ex vivo without functioning PAX7, although the number of myonuclei was reduced, suggesting less efficient myogenesis. Such PAX7-independent (possibly via the PAX7 paralog PAX3) muscle regeneration may contribute to the mild myopathic phenotype observed in MYOSCO biopsies [24].

The phenotype of *Pax7*-null mice has correlations with human pathology, with most null offspring failing to thrive (and dying in the perinatal period on certain genetic backgrounds). Those that do survive have very limited, to no, muscle regenerative capacity [[25], [26], [27], [28]], strengthening the genotype-pathology correlation in MYOSCO. A subpopulation of murine satellite cells express *Pax3* [29], which may allow some satellite cells to function.

Thus, satellite cell dysfunction underlies MYOSCO, which is a *bona fide* primary satellite cell-opathy (Table 1).

**Table 1:** Summary of Primary and Secondary Satellite Cell-opathies.

### 2.2 SELENON (SEPN1): SEPN1-myopathies

*SELENON* (formerly *SEPN1*) encodes the glycoprotein SELENOPROTEIN N that is involved in calcium homeostasis and control of oxidative stress [30,31]. Over 100 mutations spanning the *SELENON (SEPN1)* gene [32] have been associated with the autosomal recessive congenital Rigid Spine Muscular Dystrophy 1 (RSMD1; OMIM: 602771), with a prevalence of <1:1,000,000 (orpha.net) and a subset of cases of Myopathy, Congenital, With Fiber-Type Disproportion (CFTD; OMIM: 255310), jointly named SELENON (SEPN1)-myopathies [[33], [34], [35], [36]](www.LOVD.nl/SEPN1) due to the clinical overlap.

SELENON (SEPN1)-related myopathies share congenital to early onset of muscle hypotonia and weakness in neck, arm, leg and trunk muscles [[37], [38], [39]], with severity directly correlating with alterations in body mass index [40] suggesting metabolic impairment. However, levels of serum CK are usually normal [33]. Typical progressive spinal rigidity and scoliosis may contribute to further worsen neck and trunk mobility, together with affecting breathing. Indeed, severe respiratory distress is the most common clinical feature in patients, in line with diaphragmatic dysfunction, and this is already typically observed in ambulant patients. Facial weakness and mild ptosis are common, while extraocular muscle involvement is exceptional [41] (Fig. 2F). Affected muscles display variable severity across patients, possibly reflecting age of onset and type of mutation, although a direct correlation has not been fully clarified [42].

The most common histopathological feature of SELENON-myopathy is areas of myofibre hypotrophy/atrophy and, more rarely, traces of necrosis, along with foci of sarcomere disorganization (minicores), mitochondria depletion and occasional immune infiltration: overall presenting as a mild dystrophic phenotype [43,44]. CFTD patients exhibit congenital fibre-type disproportion with predominance of type I muscle fibres. A severely reduced number of satellite cells correlates with patient age, with the oldest individual showing a complete lack of PAX7-positive satellite cells, indicating a progressive loss of muscle regenerative capacity (Fig. 3C).

The satellite cell-origin of SELENON-myopathies is further strengthened by the phenotype in mouse models. Ageing *Selenon-*null mice display progressive reduction of satellite cells during repeated rounds of cardiotoxin-induced injury [45], suggesting defective satellite cell self-renewal. Inefficient regeneration in *Selenon-*null mice is accompanied by increasing fat/fibrotic infiltration and appearance of atrophic/necrotic myofibres, indicating that Selenonoprotein N deficiency leads to muscle atrophy via chronic impairment of muscle regeneration [45,46]. Injured muscles in *Selenon-*null mice fail to fully recover both mass and force after successive rounds of regeneration. That *Selenon-*null mice develop a dystrophic phenotype when muscle is stressed highlights a significant difference with the congenital onset in humans, indicating that the *Selenon-*null model does not fully recapitulate SELENON-myopathies. This also argues for other factors modulating disease severity in humans, such as genetic modifiers, environmental and/or physiological stresses.

At the molecular level, while almost undetectable in injured mouse myofibres and the majority of quiescent satellite cells, *SELENON* expression is detected transiently in 3-day regenerating muscle, concomitant with activation of resident satellite cells and peak *Pax7* expression [32]. Importantly, peak *Pax7* upregulation is blunted in *SELENON-*null satellite cells, which display enhanced proliferation ex vivo but unaltered differentiation [32,45,47]. Therefore, SELENON-myopathies join the primary satellite cell-opathy list.

### 2.3 MEGF10: myopathy, areflexia, respiratory distress, And Dysphagia, Early-Onset (EMARDD)

Murine *Multiple Epidermal Growth Factor 10 (Megf10)* was originally discovered in the same molecular screen to identify novel satellite cell markers that found *Pax7* [48], and is the homologue of human *MEGF10.* Encoding a transmembrane protein, *MEGF10* is highly expressed in adult skeletal muscle, and is involved in satellite cell proliferation and migration [49,50]. Frameshift deletions leading to translation of non-functional truncated protein, or complete mRNA loss in *MEGF10* [[51], [52], [53]] associate with a recessive congenital severe myopathy called Myopathy, Areflexia, Respiratory Distress, And Dysphagia, Early-Onset (EMARDD; OMIM: 614399). EMARDD is characterized by early onset, areflexia, respiratory distress, dysphagia and generalised hypotonia with mild elevation of serum CK [54,55] with a prevalence <1:1,000,000 (orpha.net). Notably, missense *MEGF10* mutations that affect protein folding or post-translational modifications but allow some MEGF10 function, underlie rare myopathies characterized by adult onset and minicores referred to as MEGF10-myopathies, being distinct from EMARDD [56].

Age of onset and disease severity varies in EMARDD, with weakness and hypotonia prominent in neck, arm and more distal muscles including lower limb (Fig. 2F). However, respiratory distress requiring artificial ventilation appears a universal feature [57], indicating severe diaphragm dysfunction. In addition to respiratory failure, EMARDD patients exhibit facial muscle weakness and do not achieve independent walking [57] (Fig. 2F). Muscle histology reveals a range of pathology, including variation in myofibre size (indicative of inefficient muscle regeneration), fat/fibrotic infiltration and patches of tissue necrosis [51,55,58] (Fig. 3D). While one EMARDD patient had no detectable PAX7-positive nuclei [55], other patients had normal numbers of PAX7 cells, differences likely associated with time of onset/disease duration, although contribution of other disease modifiers cannot be excluded.

How lack or loss-of-function of *MEGF10* leads to satellite cell dysfunction in EMARDD/MEGF10-myopathy is not fully understood, but loss of *Megf10* activity is likely to have a major impact on the early phases of muscle regeneration. Muscle and myofibres in the *Megf10-null* mouse appear indistinguishable from wild-type [50], but muscle regeneration is severely impacted when modelling chronic regeneration in dystrophy, providing a possible explanation for onset/phenotype variability observed in patients. Murine *Megf10-*null muscles display expanding areas of myofibre atrophy, size variation and fibrosis and variable numbers of PAX7-positive cells, resembling the progressive myopathic phenotype reported in patients. *Megf10* is highly expressed in quiescent and activated murine satellite cells [49], and the number of activated/proliferating *Pax7/Myod* double positive satellite cells is reduced in *Megf10*-null mice [50]. Dysfunctional MEGF10 could inhibit self-renewal, expansion and migration during regeneration [49], since siRNA knockdown of mouse *Megf10* results in decreased satellite cell proliferation and precocious differentiation [49,53]. Furthermore, expression of c.2320T > C (associated with severe pathology [55]) in murine myoblasts phenocopies lack of MEGF10, with impaired proliferation and migration [53]. Notably, MEGF10 interacts with Notch1, whose signalling is crucial during the quiescence to activation transition [59,60], further suggesting contribution of satellite cell dysfunction to EMARDD onset and progression. EMARDD is a primary satellite cell-opathy (Table 1).

Recently, pathogenic mutations in *JAG2* (*JAGGED2*) that are predicted to impair protein function have been identified in a cohort of patients. Their muscular dystrophy resembles EMARDD, including facial weakness, respiratory complications and mildly elevated CK level [61]. *JAG2* encodes for a NOTCH3 ligand that contains several EGF repeats, similar to MEGF10 and other Notch ligands [62], and Notch3 is involved in satellite cell self-renewal in mouse [63]. Knockdown of *Jag2* in murine muscle cells reduced Notch1 activation, concomitantly leading to a severe reduction in *Megf10* expression. This suggests that the molecular mechanism of the muscular dystrophy caused by *JAG2* mutations overlaps with that underlying EMARDD, and also argues for *JAG2* as a putative gene modifier in *MEGF10*-myopathy. Notably, patients affected with JAG2-myopathy also display severe reduction of PAX7, concomitant with reduced expression of its target gene *MYF5*, suggesting a dysfunctional satellite cell niche.

### 2.4 CAPN3: Limb-Girdle Muscular Dystrophy Recessive 1 (LGMDR1)

The fourth myopathogene with involvement in satellite cell biology is *CAPN3* encoding CALPAIN 3, a member of the calpain family of calcium-dependent papain-like proteases with predominant expression in skeletal muscle.

Loss of function mutations in *CAPN3* have been linked with the autosomal recessive Limb-Girdle Muscular Dystrophy Recessive 1 (LGMDR1; OMIM: 253600 (formerly LGMD2A)) the most common form of LGMD worldwide, accounting for one third of all recessive cases with a prevalence of 1–9:100,000 (orpha.net). Variants leading to less common autosomal dominant forms with later onset and milder phenotype also occur (LGMDD4; OMIM: 618129) [[64], [65], [66], [67]].

Onset of LGMDR1 ranges from childhood to adulthood (second/third decade of life) and the rate of disease progression also varies, mostly correlating with the type of mutation in *CAPN3*. Null mutations result in a more homogeneous pathology, with early onset and rapid course [68]. Over 500 pathogenic *CAPN3* mutations have been described, including nonsense, frameshift, deletions or duplications (www.LOVD.nl/CAPN3), most likely leading to transcript degradation through non-sense mediated decay and absence of CAPN3 [69,70]. Some patients bear *CAPN3* mutations that impair CALPAIN 3 function, but not quantity [71,72].

Clinically, CAPN3-deficiency presents the classical LGMD symmetric progressive weakness and atrophy of the musculature of the shoulder/upper arm and pelvic girdle/upper leg, together with wasting of trunk muscle [67,68] (Fig. 2F). Serum CK levels are markedly elevated [73]. Respiratory distress is extremely rare, suggesting relative sparing of the diaphragm [74]. Scapular winging or calf hypertrophy may be present, but significant facial weakness is absent, which helps in the differential diagnosis with facioscapulohumeral (FSHD), Becker or Duchenne muscular dystrophies [75,76].

Muscle biopsies display myofibre size variation resulting both from hypotrophy and atrophy, where the latter seems to reflect disease duration, but hypoplasia (reduced myofibre number) is reported [71]. Formation of lobulated myofibres, lacking proper cytoarchitecture, is also considered a major pathological hallmark. Dystrophic areas are characterised by inflammatory infiltrates, along with fat/fibrotic tissue replacement indicating necrosis and poor regeneration [77]. Strikingly, a significant increase in satellite cells occurs in muscle biopsies, suggesting that inefficient regeneration may result from altered satellite cell dynamics or function. Intriguingly, altered satellite cell numbers is fibre-type dependent, with type I having less satellite cells compared to type II myofibres, opposite of the situation in healthy muscle [78].

Is LGMDR1 a satellite cell-opathy? Unlike *PAX7*, *MEGF10* and *SELENON*, the link between the molecular function of *CAPN3* and satellite cell homeostasis is unclear. Through its proteolytic activity, CALPAIN 3 contributes to the homeostatic turn-over of sarcomere components, such as TITIN, FILAMIN C and TALIN and its loss-of-function has been associated with delayed myofibrillogenesis and defective sarcomere assembly both in vitro and in mouse models, so CALPAIN 3 is crucial in mature myofibres [[79], [80], [81]]. Expression of *CAPN3* during human muscle development is relatively late, following myotube formation and limb muscle innervation at 8 weeks of embryogenesis [82]. Crucially, Calpain 3 regulates generation of reserve cells, quiescent satellite cells generated in vitro during myoblast differentiation [83] through degradation of MYOD, an important regulator of satellite cell activation [2]. Moreover, *Capn3*-null mouse muscle shows altered mTOR signalling, which marks the onset of satellite cell activation [84,85]. Thus, lack of CALPAIN 3 likely affects the efficiency of satellite cell activation, in addition to its later function in myofibre maintenance, making LGMDR1 a likely secondary satellite cell-opathy.

### 2.5 MYOD: myopathy, congenital, With Diaphragmatic Defects, respiratory insufficiency, And Dysmorphic Facies (MYODRIF)

MYOD belongs to the myogenic regulatory factor family of transcription factors (MRFs), and its roles in muscle development and satellite cells are well established [2,86]. Strikingly, despite extensive literature demonstrating its essential functions in satellite cells, MYOD was not a member of the 30 genes we derived from the GOSC, nor in the 2020 gene table of neuromuscular disorders [15]. However, pathogenic *MYOD* variants are associated with the extremely rare Myopathy, Congenital, With Diaphragmatic Defects, Respiratory Insufficiency, And Dysmorphic Facies (MYODRIF; OMIM: 618975) [[87], [88], [89]]. Despite extensive exome sequencing, the exceptionally limited number of cases suggests that loss-of-function MYOD variants are more usually incompatible with life.

The first loss-of-function variant in MYOD was reported in three siblings that suffered from decreased foetal movement, facial dysmorphisms and poor lung development, leading to perinatal death [89]. A later case reported severe respiratory failure concomitant with muscle weakness, hypotonia and motor delay in an 8-year-old patient bearing a biallelic truncating mutation at the exon 2/3 junction [87]. These symptoms and myopathic hallmarks in muscle biopsy, were then confirmed in two siblings bearing a frameshift mutation causing premature termination of *MYOD* mRNA [88]. All reported patients had abnormal diaphragm function and facial dysmorphism resembling clinical features of MYOSCO, EMARDD and RSMD1 (Fig. 2F), but generally normal serum CK levels [88]. Inactivation of *Myod* in mouse has little effect on muscle formation and adult musculature except for an increase in satellite cell numbers [90] but regeneration is delayed [91] and satellite cells differentiate poorly ex vivo [92]. Thus, although assessment of satellite cell number/status is unavailable for MYODRIF, the known function of MYOD in satellite cells [2,83] and shared patho-phenotype with known primary satellite cell-opathies (Table 1) places MYODRIF in the category of a primary satellite cell-opathy.

### 2.6 MYOMAKER (MYMK): Carey-Fineman-Ziter Syndrome (CFZS)

MYOD, together with its MRF family member MYOGENIN, orchestrate expression of genes required for terminal myogenic differentiation [2]. Differentiating myoblasts fuse together or to myofibres, contributing new myonuclei, so fusion-deficient myoblasts would directly impair muscle growth and repair. MYOMAKER (MYMK/TMEM8C) is an essential member of the cell fusion machinery, whose expression is directly regulated by MYOD and MYOGENIN across vertebrates [[93], [94], [95], [96]].

Although absent from our GOSC list, *MYMK* is a myopathogene [15]. Pathogenic missense *MYMK* variants are associated with the congenital myopathy Carey-Fineman-Ziter Syndrome (CFZS; OMIM: 254940) [97,98]. Disease presents as static generalised hypotonia and weakness. Facial weakness and respiratory distress, likely due to weak diaphragm, are often present [99] and resemble MYOSCO, EMARDD (Table 1) and MYODRIF. Muscle biopsies from quadriceps display limited myopathic features but marked myofibre hypertrophy, likely arising from dysregulated, rather than a lack of, myoblast fusion, and generalised fibre-type disproportion that resembles SELENON-deficiency [[99], [100], [101]] (Fig. 3E). Notably, several patients displayed increased serum CK [98], suggesting that other muscle groups may display more severe myopathy, with the quadriceps being relatively spared at time of biopsy.

Although satellite cell status in CFZS patients is unknown, *MYMK-*null human immortalised myoblasts display severe fusion deficits, closely resembling the phenotype in animal models [94], where *Mymk-*null mouse and zebrafish show severely defective muscle formation with overabundance of mononucleated myofibres. Indeed, human pathology is probably mitigated by residual MYMK function and/or *Mymk*-independent pathways that allow some myonuclear accretion, as observed in *Mymk-*null zebrafish [93,96,102]. While *Mymk* is indispensable in fusing murine myoblasts, myofibre-specific *Mymk* depletion does not hamper regeneration in mature muscle, indicating that loss-of-function mutation(s) in *MYMK* specifically impedes satellite cell function [103,104]. Since CFZS myopathy resembles MYOSCO, RSMD1 and EMARDD, we categorize CFZS as a primary satellite cell-opathy (Table 1).

MYOMIXER (encoded by *MYMX*) is also essential for myoblast fusion [122] and recently siblings with a homozygous hypomorphic *MYMX* mutation have been identified that have a disorder with similarities to CFZS (personal communication: Eric N. Olson, UT Southwestern Medical Center).

### 2.7 Could mutations in other GOSC genes cause satellite cell-opathies?

The remaining 26/30 genes in our GOSC do not overlap with the 116 (or complete 170+) myopathogenes [15], so mutations are not currently associated with muscular dystrophies, congenital muscular dystrophies, congenital myopathies or distal myopathies (Fig. 2D and E). However, some of these remaining 26 genes in the GOSC may also be myopathogenes, and thus cause satellite cell dysfunction when mutated.

*PAXBP1* (*Pax3/7 binding protein1*) connects PAX7 to chromatin methylation machinery, allowing expression of the PAX7 transcriptome. Knockdown of mouse *Paxbp1* reduces myoblast proliferation [105], suggesting that loss-of-function variants in human should phenocopy MYOSCO. Indeed, a pathogenic variant of *PAXBP1* is associated with developmental delay, muscle hypotonia and weakness, but conclusive genotype-phenotype correlation is currently unavailable [106].

*Mylk2* is expressed in satellite cells and correlates with histone deacetylation. Pathogenic mutations in *MYLK2* are associated with cases of familial hypertrophic cardiomyopathy (OMIM: 192600) [107], but whether there are also subtle effects on satellite cell function should be investigated.

*Akirin1* participates in regulation of chemotactic signalling during muscle regeneration, potentially through upregulation of Myod via IGFII, but is inhibited by Myostatin [108,109]. *Akirin1* is upregulated during satellite cell activation and is crucial for macrophage function [108], so its loss of function in human could lead to a muscular dystrophy/myopathy.

Loss of Myostatin function results in muscle hypertrophy in human [110], mouse [111,112] and fish [113], with *Mstn-*null mouse and chick displaying increased satellite cell number [114,115]. Gain-of-function mutations in human may manifest in muscle disease.

## 3 Secondary satellite cell-opathies

Primary satellite cell-opathies are caused by perturbed satellite cell function, and so only have indirect effects on muscle fibres. We define secondary satellite cell-opathies as conditions that arise from mutations in genes involved in both satellite cell and myofibre function, potentially leading to chronic injury/repair cycles and gradual degradation of the muscle microenvironment and function (Table 1). Interestingly, only *CAPN3* in the GOSC was also a myopathogene causing a secondary satellite cell-opathy. There are further clear candidates for myopathogenes that cause secondary satellite cell-opathies in the literature. Exemplars include Muscular Dystrophy, Congenital, Lmna-Related (MDCL; OMIM: 613205), Emery-Dreifuss dystrophy 2 (EDMD2 (formerly LGMD1B); OMIM: 181350), Muscular Dystrophy, Congenital Merosin-Deficient, 1a (MCD1A; OMIM: 607855 and LGMDR23; OMIM 618138); and FSHD (OMIM: 158900) (Table 1).

Activated satellite cells undergo several rounds of cell cycle, requiring continuous nuclear disassembly and reassembly during mitosis, and when fusing, myoblasts must rearrange their nuclear envelope within the myofibre [116]. Hence, mutations in genes encoding proteins that control nuclear architecture and integrity could impair the regenerative process at multiple levels. *LMNA* encodes mainly for Lamin A and Lamin C, structural components of the nuclear lamina, and also present in the nucleoplasm to modulate nuclear integrity and chromatin organisation [117]. *LMNA* is expressed in both satellite cells and myonuclei and mutations are associated with the heterogenous group of disorders called laminopathies. Two autosomal dominant laminopathies involve skeletal muscles. MDCL and EDMD2 show overlapping features, with MDCL congenital and EDMD2 often having childhood onset [118]. Both conditions present as muscle weakness/contractures and atrophy, phenocopying LGMD, with variable rates of progression, and often accompanied by cardiac dysfunction [119]. CK levels are mildly to moderately increased in MDCL. Muscle biopsies show irregular myonuclear shape as well as chromatin disorganization, in line with Lamin A/C function in nuclear structure and epigenetic organisation [120]. Nuclear shape and chromatin density are also altered in satellite cells, and the number of PAX7-containing satellite cells is increased in EDMD2 patients compared to unaffected controls, but mechanisms remain ill defined [121]. *Lmna-*null murine satellite cells have delayed activation and slow proliferation, concomitant with decreased *MYOD* accumulation [121] that could explain supernumerary satellite cells in patients. *Lmna-*null myoblasts also differentiate poorly, and overexpression of pathogenic missense Lamin variants in healthy myoblasts reduces both proliferation and fusion ability despite increased expression of both fusogens *Mymk* and *Mymx* [122], suggesting that Lamin A/C deficiency alters satellite cell function. Fewer myonuclei are found in *Lmna-*null mice, indicating poor myoblast fusion, correlating with altered satellite cell number [94,[123], [124], [125], [126], [127]].

Laminins stabilise the myofibre-extracellular matrix connection (ECM) through the Dystrophin-Associated Protein Complex (DAPC), but are also involved in establishing satellite cell polarity during the initial division phase [128]. Laminin Subunit Alpha 2 or 211 (merosin) is the predominant isoform in the basal lamina, with over 350 pathogenic *LAMA2* mutations listed (www.LOVD.nl/LAMA2). Loss of *LAMA2* causes Muscular Dystrophy, Congenital Merosin-Deficient, 1a (MDC1A; OMIM: 607855), while partial *LAMA2* deficiency results in LGMDR23 (OMIM: 618138). MDC1A patients suffer muscle weakness and hypotonia, pronounced in upper and lower limbs, with onset at birth or within the first six months. Respiratory distress due to trunk/diaphragm weakness is reported in most MCD1A patients [129]. Serum CK levels are also elevated. Histological analysis shows dystrophic features such as necrosis, muscle fibre size variation and fat/fibrotic infiltrates indicative of both myofibre instability and poor regeneration. Several *Lama2-*null mouse models demonstrate a dystrophic phenotype with *in utero* onset [130,131], characterized by poor regeneration, immature myofibres and cell death [132]. *Lama2-*null display foetal loss of Pax7-containing satellite cells and increase of both *Akirin1* and *Myod* expression suggesting an inability to maintain the stem cell niche. In accordance, *LAMA2* is expressed in both quiescent mouse and human satellite cells [133]. Moreover, murine *Lama2*-null myoblasts proliferate and fuse poorly whereas LAMA2-CMD myoblasts display elevated cell death [[134], [135], [136]] congruent with inefficient regeneration in null mice and CMD muscles.

Muscle disorders with a generally later onset such as FSHD also fit the category of secondary satellite cell-opathies (Table 1). Substantial deletion of 3.3.kb D4Z4 repeats in the sub-telomeric region of chromosome 4q35 [[137], [138], [139]] releases epigenetic repression, in FSHD1. This genomic change allows the open reading frame in the last D4Z4 unit on permissive haplotypes to transcribe the ‘toxic’ transcription factor DUX4 in muscle fibres and myoblasts [[140], [141], [142]]. As the name Facioscapulohumeral muscular dystrophy implies, facial, upper limb girdle, trunk and lower limb musculature are initially affected with a descending progression, with CK levels normal to elevated [143]. In line with the dystrophic phenotype, beside myofibre degeneration and atrophy, patient biopsies also display low levels of regeneration, suggestive of poor satellite cell function [144]. In fact, transcriptomic analysis reveals perturbed myogenesis [145] and repression of PAX7-target gene expression that correlates with disease progression [143,146,147], implying that DUX4/PAX7 interactions may contribute to FSHD pathogenesis [148]. This hypothesis is supported by observations that both PAX7 and DUX4 inhibit the ability of the other to activate their transcriptional target genes [146]. Furthermore, PAX7 overexpression protects mouse myoblasts from DUX4 toxicity, although such an effect is yet to be shown in human cells [149].

Recent evidence suggests that Duchenne muscular dystrophy (DMD; OMIM: 310200) may also be a secondary satellite cell-opathy. DMD arises from mutations in the X-linked *Dystrophin* (*DMD*) gene encoding DYSTROPHIN, an essential component of the DAPC that stabilises the myofibre by connecting the contractile apparatus to the ECM [150]. There are over 5000 *DMD* pathogenic mutations (www.LOVD.nl/DMD), with most decreasing/eliminating DYSTROPHIN so altering DAPC assembly and impairing myofibre integrity during contraction [[151], [152], [153]]. DMD is not congenital, with normal muscle formation and postnatal growth, but manifests in early childhood with a rapidly progressive weakness and wasting in the musculature of the neck, trunk and arm, descending to lower limbs as disease advances. Muscle biopsies display extensive necrosis, fibrosis and fat infiltrations resulting from chronic rounds of muscle degeneration and regeneration with increased number of PAX7-positive satellite cells reported initially [154]. *Dystrophin* is expressed by rodent satellite cells [155] and during activation, where it is reported to contribute to asymmetric cell division to maintain the stem cell niche [156]. *Dmd* depletion also perturbs mouse myoblast proliferation and reduces terminal differentiation [156]. Although muscle regeneration is robust in *mdx* mice (containing a nonsense mutation in *Dmd* exon 23) [157], it can worsen over time to resemble aspects of human disease. Becker muscular dystrophy (BMD; OMIM: 300376) is also associated with mutations in DMD, but they tend to encode a partially functional DYSTROPHIN protein and so BMD is generally milder than Duchenne, and could also be considered a secondary satellite cell-opathy.

Muscle disorders with an adult onset that also demonstrate defects in satellite cell function also fit the category of secondary satellite cell-opathies. Examples include Myotonic Dystrophy 1 (DM1; OMIM: 160900) [158,159] and Oculopharyngeal Muscular Dystrophy (OPMD; OMIM: 164300) [160,161].

## 4 Non-satellite cell-opathy neuromuscular disorders

Certain neuromuscular disorders are caused by mutations in genes that are unlikely to affect satellite cell function. In such neuromuscular disorders, muscle fibres are constantly damaged, but the repair/regenerative response is not directly affected. However, regenerative myogenesis may become compromised by an increasing hostile muscle microenvironment. Neuromuscular disorders with underlying mutations in myopathogenes such as Myosin genes (*MYH7*, *MYH3*, *MYH8*, *MYH2*, *MYL2, MYL1*) fit the remit of neuromuscular disorders that are not primary or secondary satellite cell-opathies [15]. Interestingly, a rare neonatal-onset congenital myopathy Myopathy, Proximal, With Ophthalmoplegia (MYPOP; OMIM: 605637) associated with a novel heterozygous variant in *MYH2,* presents with classical muscle weakness accompanied by dysmorphic features and respiratory problems [162].

Similar to myosins, pathogenic mutants of proteins contributing to maintain the contractile properties of the myofibre should not impact satellite cells. Mutations in the sarcomeric component MYOPALLADIN *(*encoded by *MYPN*) causes several cardiomyopathies and a congenital form of slowly progressing nemaline myopathy with myofibre size variation and evident atrophy termed Nemaline Myopathy 11, Autosomal Recessive (NEM11; OMIM: 617336) [163]. MYOPALLADIN is crucial for sarcomeric integrity during muscle contraction. *Mypn-*null mice are more prone to exercise-induced injury but show unaltered regeneration potential [164]. Likewise, MYOTILIN *(*encoded by *MYOT*) serves as a structural component of the sarcomere, and *MYOT* mutations have been identified as the most common underlying cause of Myopathy, Myofibrillar, 3 (MFM3; OMIM: 609200) with no satellite cell dysfunction reported [165].

As seen with DYSTROPHIN though, since examination of genes encoding proteins associated with muscle fibre structure, integrity and contraction are often neglected when studying satellite cells, putative effects of mutant versions on the stem cell population remains a possibility in some Non-satellite cell-opathy neuromuscular disorders.

## 5 One size does not fit all

Finer classification of muscle disease is an ever-growing field. High-throughput technologies have contributed to stratify muscle conditions, discerning the molecular origin of conditions with overlapping phenotypes [166]. However, a better standard for grouping clinical, cellular and molecular aspects is needed. Although useful, exploiting Gene Ontologies (GOs) to address genotype-phenotype correlations does not fully describe the cellular origin of a specific muscle condition. As discussed, despite having well-recognised functions in satellite cells, *MYOD* and *MYMK* are not included in our novel GOSC (Fig. 2C and D), demonstrating that current ontological annotation is not a single parameter to discern satellite cell-opathies. Conversely, *MYOG* is annotated in GOSC, decreases rapidly during mouse satellite cell activation, and the lack of MYOGENIN leads to dramatic accumulation of fusion-deficient satellite cells in zebrafish, but no pathogenic variants in human have been reported [167,168]. In contrast to *MYOD*, while *CAPN3* can be found in GOSC, indication of CALPAIN 3 function in satellite cells is limited to in vitro manipulation [83]. Therefore, multiple aspects must be considered to identify further putative satellite cell-opathies.

## 6 Tool for uncovering satellite cell-opathies across known muscle conditions

How to identify further *bona fide* satellite cell-opathies among inherited myopathies and dystrophies? Having established a limitation to using our current GOSC to predict satellite cell dysfunction arising from known pathogenic variants (Fig. 2), we devised a multimodal strategy to highlight potential satellite cell dysfunction due to a given myopathogene.

**Fig. 4:**
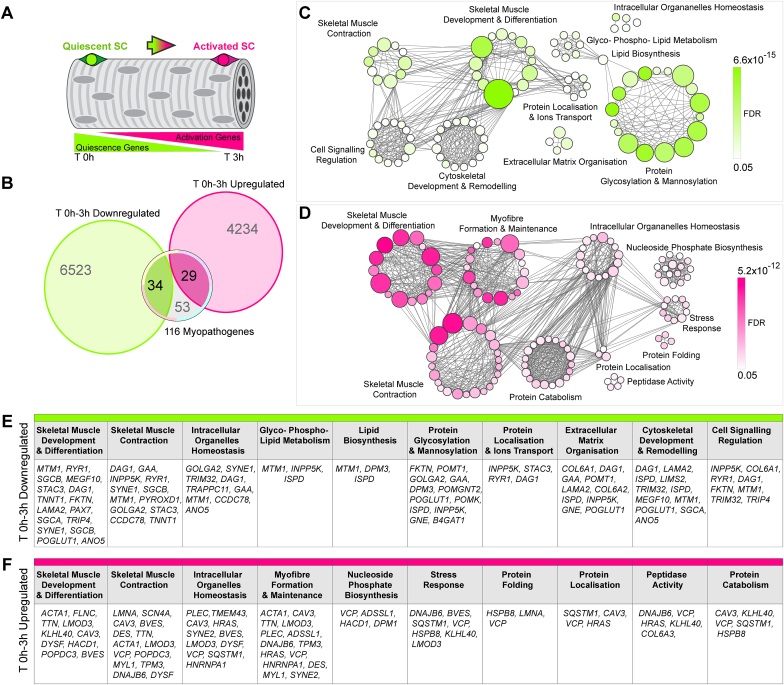
**Ontological analysis of the 63 myopathogenes differentially expressed during early satellite cell activation**. **A.** Schematic of quiescent/activated satellite cell gene-sets [168] used to explore involvement of the 116 myopathogenes in satellite cell biology. Diagram shows transcriptomic changes occurring during early murine satellite cell activation, where genes involved in quiescence (green, T 0h) are likely to be downregulated in parallel with upregulation of genes supporting satellite cell activation (magenta, T 3h). **B.** Venn diagram illustrating overlap between significant differentially expressed genes (downregulated, green and upregulated, magenta) during the first 3 hours (T 0h vs T 3h) of satellite cell activation (A) and the 116 myopathogenes [15]. **C** and **D.** Bubble plots demonstrating networks of enriched Gene Ontologies (GOs), clustered according to main biological processes, of differentially expressed myopathogenes during early satellite cell dynamics. **C.** Main biological processes enriched in downregulated myopathogenes (green). **D.** Main biological processes enriched in upregulated myopathogenes (magenta). GOs (bubbles) were retrieved using Metascape (metascape.org; [240]) and Panther Gene Ontology (geneontology.org; [241]) and visualised using Cytoscape (cytoscape.org; v3.8.2; [242]). Layout parameters were optimised for presentation. Bubbles are coloured based on False Discovery Rate (FDR) values and size is proportional to number of genes within specific GO terms, grey lines represent genes shared across different GOs. **E** and **F.** Differentially expressed (down, green **E**; up, magenta **F**) satellite cell-myopathogenes annotated within main biological process (GOs clusters).

1. To infer a satellite cell-contribution of a myopathogene, we used a recently published dataset of genes differentially expressed within the first 3 hours of adult murine satellite cell activation [168] to screen the list of 116 myopathogenes (Fig. 4, Fig. 5).

**Fig. 5:**
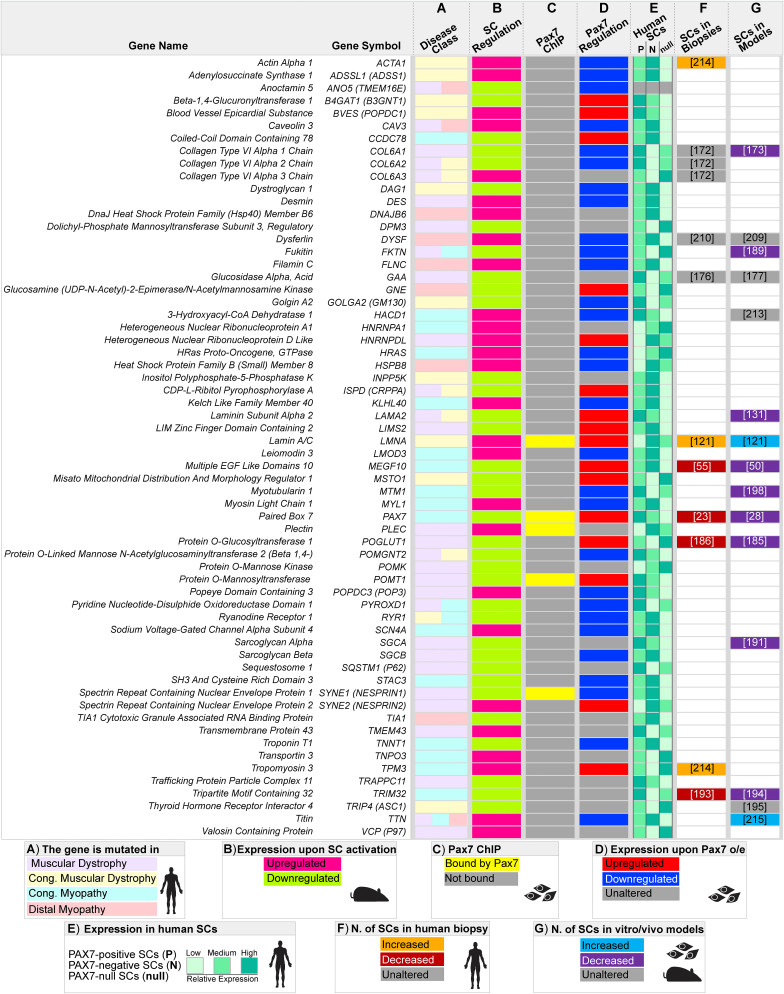
**Summary of myopathogenes associated with satellite cell function**. Heatmap depicting the characteristics of the 63 myopathogenes associated with satellite cells. **A.** Mutation in specific neuromuscular disease class/es [15]. **B.** Differential expression during early satellite cell (SC) activation in mouse [168]. **C.** Binding by Pax7 in chromatin immunoprecipitation (ChIP) in mouse myoblasts [169]. **D**. Differential regulation in response to Pax7 overexpression (o/e) in mouse myoblasts ([169]; LogFold Change≥ 0.5 or ≤ −0.5). **E**. Expression in PAX7-positive, PAX7-negative or PAX7-null human satellite cells ([24] reported as low, medium or high, based on counts per million (cpm). **F**. Effect of the myopathogene mutation on the number of satellite cells in patients with citation. **G.** Effect of the myopathogene mutation on satellite cells in *in vivo/vitro* models with citation. Sample type and species source of data from human, mouse or cultured cells are depicted by icons in the key.
2. As PAX7 is recognised as a master regulator of satellite cells, we next assessed Pax7-driven regulation of selected myopathogenes using transcriptomic changes and Pax7-DNA binding in murine embryonic stem cells (ESCs) upon Pax7 upregulation [169] (Fig. 5).
3. To refine regulation of selected myopathogenes by human PAX7 we interrogated a publicly available RNAseq dataset of wild-type PAX7-positive and PAX7-negative satellite cells isolated from healthy human biopsies and PAX7-null satellite cells from a MYOSCO patient [24] (Fig. 5).
4. Where data is available, we also considered whether satellite cells are affected in the associated human disease and animal models.

## 7 Genes that may cause novel satellite cell-opathies

Our comparison identified that during murine satellite cell activation (Fig. 4A), 34/116 (29%) myopathogenes were downregulated, whereas 29/116 (25%) were upregulated (Fig. 4B). Thus 63 myopathogenes may function in human satellite cell quiescence or activation, and so mutations may affect satellite cell function to contribute to pathology in muscle disorders (Fig. 4B). As expected, GOSC genes *PAX7* and *MEGF10* are downregulated upon exit from quiescence (Fig. 4C,E). Expression of 46/63 (73%) differentially regulated myopathogenes oscillate upon Pax7 induction (Fig. 5), but only 5 appear to be bound by Pax7 namely *LMNA*, *PAX7*, *PLEC*, *POMT1* and *SYNE1* (Fig. 5) indicating most myopathogenes are not direct PAX7 target genes. To understand potential pathomechanisms involved in satellite cell dysfunction, we interrogated the selected satellite cell-expressed myopathogenes using GO analysis to evaluate their contribution to specific biological processes. Some biological functions appear specific for either downregulated or upregulated satellite cell-expressed myopathogenes, such as “Extracellular matrix organisation” or “Myofibre formation and maintenance” respectively (Fig. 4C - F). Others however, such as “Skeletal muscle development and differentiation” and “Skeletal muscle contraction”, notionally characteristic of differentiating/differentiated cells, appear to represent a subset of myopathogenes in both groups (Fig. 4C - F). We next discuss evidence supporting satellite cell dysfunction in a number of muscle conditions, by considering the biological process in which a particular myopathogene is involved (Fig. 4E and F), and how the particular biological role sustains satellite cell homeostasis.

### 7.1 Extracellular matrix

ECM and cytoskeleton organisation were enriched in the subset of myopathogenes downregulated as satellite cells leave quiescence (Fig. 4C). Since ECM supports the regeneration process and influences satellite cell activity [170,171] it is logical that loss-of-function in any of these genes could directly lead to satellite cell dysfunction. The presence of *LAMA2* in this selection is encouraging given its role in MCD1A. Expression of *LAMA2* is downregulated as satellite cell activate, corroborating its cell-autonomous need in satellite cell homeostasis (Fig. 4). In line, *LAMA2* is upregulated in response to ectopic mouse Pax7, and it is decreased in human satellite cells lacking PAX7 (Fig. 5).

COLLAGENS VI (encoded by *COL6A1* and *COL6A2*) mutations associate with myofibre fragility in Bethlem myopathy 1 (BTHLM1:OMIM: 158810) and Ulrich congenital muscular dystrophy 1 (UCMD1; OMIM: 254090) [172] in which weakness in facial, trunk and more proximal muscle accompany a respiratory insufficiency which worsens over time. Our analysis suggests that satellite cell behaviour could be affected. *Col6a1-*null mouse muscle has fewer satellite cells and they activate poorly, leading to both inefficient self-renewal and regeneration. Reduced ability of *Col6a1-*null satellite cells to engraft wild-type muscle implies a cell-autonomous need [173]. Likewise, UCMD1 muscles also display limited regenerative capacity, although with normal steady-state satellite cell numbers [172]. *COL6A3* expression displays the opposite trend compared to its paralogs, being upregulated during satellite cell activation and strongly induced in PAX7*-*null satellite cells (Fig. 4, Fig. 5). Interestingly, satellite cell quiescence is partially maintained through signalling via another Collagen (V) [59] and Collagen-derived dipeptide oral administration improves muscle regeneration in mice [174], strengthening the hypothesis that loss of Collagen(s) expression/function may impede satellite cell activity.

*GAA* encodes for lysosomal α-glucosidase, essential for degradation of glycogen to glucose in lysosomes, which is mutated in Pompe disease/Glycogen Storage Disease II (GSD2 (formerly LGMD2V); OMIM: 232300). Its classification as a LGMD or metabolic disease is unclear, as muscle biopsies show limited fibrosis and inflammation, but damage to myofibres from lysosomal glycogen accumulation [175]. Despite signs of myopathic histology, GSD2 biopsies display normal numbers of satellite cells and indistinguishable activation/proliferation (as assessed with MYOD/Ki67) compared to controls. In contrast, *Gaa-*null mice have insufficient satellite cell activation and muscle regeneration during disease progression [[176], [177], [178]], so loss of lysosomal α-glucosidase function compromises murine satellite cell activity. Importantly, trunk and diaphragm weakness often cause respiratory failure.

Therefore, evidence of satellite cell dysfunction suggests UCMD1, BTHLM1 and GSD2 as putative satellite cell-opathies.

### 7.2 Glycosylation

Several ECM/cytoskeletal components require post-translational modification for proper function. Our analysis indicates that a proportion of myopathogenes, downregulated as satellite cells exit quiescence, contribute to protein and lipid glycosylation/mannosylation and cytoskeletal remodelling in satellite cells (Fig. 4C,E). Indeed, glycosylation is critical for cell physiology and its pathological involvement is well known in muscle diseases [179,180] as its alteration enhances muscle vulnerability during contraction and influences satellite cell function [181].

α-DYSTROGLYCAN and β-DYSTROGLYCAN (encoded by *DAG1)* are parts of the DAPC that provides myofibre stability and α-DYSTROGLYCAN is subjected to extensive glycosylation that regulates interaction with its ligands. α-DYSTROGLYCAN hypoglycosylation is associated with dystroglycanopathies that are classified depending on whether the causative mutation affects α-DYSTROGLYCAN itself (primary) or genes encoding for α-DYSTROGLYCAN-glycosylating enzymes (secondary or tertiary) [182]. Severity of the clinical presentation varies largely across dystroglycanopathies, which include Walker–Warburg syndrome (WWS), muscle-eye-brain disease (MEB), Fukuyama congenital muscular dystrophy (FCMD) and Fukutin related dystrophy. Genetic diagnosis means that these conditions are now classified as Muscular Dystrophy-Dystroglycanopathy (Congenital With Brain And Eye Anomalies), Type As (MDDGA)). Nearly all patients display reduced α-DYSTROGLYCAN glycosylation in muscle biopsies and are initially diagnosed with classical congenital muscular dystrophic symptoms associated with respiratory difficulty, suggesting weakness and wasting in both trunk muscles and diaphragm [183]. Notably, several congenital muscular dystrophies also present abnormal eye movements and strabismus, implying poor function of facial/eye muscles.

Our analysis shows that several myopathogenes associated with secondary or tertiary dystroglycanopathies (*POMT1*, *FKTN*, *POGLUT1*, *POMK*, *POMGNT2*, *ISPD* and *B4GAT1*) are downregulated during satellite cell activation (Fig. 4C,E) [184]. Interestingly, *POMT1* is a direct Pax7-target gene and strongly reduced in PAX7*-*null satellite cells (Fig. 5).

Loss of *POGLUT1* (*protein O-glucosyltransferase1*) causes LGMDR21 (OMIM: 617232) and alters α-DYSTROGLYCAN glycosylation, with a severe reduction of satellite cells and *PAX7* expression blunting muscle regenerative capacity [185,186]. Moreover, induction of murine *Pax7* results in accumulation of *Poglut1*, *B4gat1* and *Ispd*, possibly indirectly as they are not bound by Pax7, although their expression differs in human PAX7-null satellite cells (Fig. 5). Intriguingly, lack of POGLUT1 also dysregulates Notch1 glycosylation [186]. In turn, disruption of Notch1 signalling in murine satellite cells phenocopies aspects of POGLUT1-deficiency, resulting in a dystrophic phenotype with reduction of Pax7-positive cells and decreased *Pax7* expression, suggestive of defective self-renewal [187]. Thus LGMDR21 shares pathogenic features with EMARDD [55,57,58] and JAG2-myopathy [61], and can be considered a satellite cell-opathy.

Similarly, since myofibre-specific depletion of *Fukutin* (*Fktn*) in mice results in a mild myopathy compared to the more severe muscle wasting reported when the *Fktn* gene is specifically depleted in satellite cells, it is plausible that satellite cell dysfunction contributes to MDDGA4 (Fukuyama/FKTN related dystrophy (OMIM: 253800) [188,189]. Although satellite cell analysis on MDDGA4 human muscles has not been reported, since FUKUTIN contributes to α-DYSTROGLYCAN glycosylation and *Fktn* levels respond to Pax7 abundance (as does *Pomgnt2* (Fig. 5)), supports the hypothesis that satellite cell dysfunction could contribute to most dystroglycanopathies. In line with this, DYSTROGLYCANS contribution as a cell-signalling scaffold [156] is also retrieved by our analysis in activating satellite cells (Fig. 4F).

Finally, SARCOGLYCANS are core DAPC organisers, mutated in a subgroup of LGMDs termed Sarcoglycanopathies [190]. Mutations in *SGCA* cause LGMDR3 (OMIM: 608099) and while satellite cell status in human biopsies is unknown, *Sgca-*null mouse myoblasts show reduced proliferation and poor muscle engraftment, although there are normal numbers of satellite cells in *Sgca-*null muscle [191].

### 7.3 Cell Signalling Regulation

Along with *DAG1* and *FKTN*, *TRIM32* is also annotated in the biological process “Cell Signalling Regulation”. Mutations in *TRIM32* are associated with LGMDR8 (formerly LGMD2H; OMIM: 254110), that usually has a childhood onset and progressive classical LGMD weakness and wasting, as well as facial weakness and respiratory distress [192]. Muscle biopsies show dystrophic changes such as centralised nuclei and degenerating myofibres, in parallel with a severe reduction in satellite cells, likely due to premature senescence and dysregulated autophagy [193]. TRIM32-deficient human myoblasts also display poor proliferation and differentiation that possibly contributes to limiting regenerative potential [193]. Interestingly, *Trim32-*null mouse muscle subjected to an atrophy/regrowth regime displays a progressively loss of satellite cells [194], indicating defective maintenance of the satellite cell pool.

*TRIP4* is mutated in Muscular Dystrophy, Congenital, Davignon-Chauveau Type (MDCDC; OMIM: 617066); and its knockdown results in blunted myogenic differentiation. While satellite cell number is unaltered, PAX7-expressing human satellite cells have increased *TRIP4* levels compared to PAX7-null, in line with *TRIP4* accumulation in murine quiescence satellite cells [195] (Fig. 5), hence specific examination of satellite cell number in human MDCDC is needed. In fact, patients experience generalised hypotonia and muscle wasting, but also respiratory insufficiency due to muscle weakness, implying diaphragm dysfunction [195].

MYOTUBULARIN (encoded by *MTM1*) is a phosphoinositide phosphatase that participates in several biological processes (Fig. 4C,E) whose mutations results in Myopathy, Centronuclear, X-Linked (CNMX, or XLMTM/MTM1; OMIM: 310400). This severe congenital myopathy exhibits weakness in both facial and neck muscles, and early respiratory failure is often fatal [196]. Our expression analysis shows downregulation of *Mtm1* during murine satellite cell activation, but negative regulation to PAX7 accumulation in human satellite cells. MYOTUBULARIN-deficient biopsies display satellite cell reduction, similar to *Mtm1-*null mouse muscles in which satellite cell number declines with age and parallels disease progression [197,198]. Intriguingly, *Mtm1-*null myoblasts show reduced proliferation, limited engraftment in wild-type muscle and increased cell death, but can fuse efficiently. Thus, defective satellite cell pool maintenance underlies CNMX pathogenesis [198].

It would seem that LGMDR8 and CNMX, and probably MDCDC, can be classified as satellite cell-opathies.

### 7.4 Autophagy and unfolded protein response mechanisms

Exit from quiescence is accompanied by increased protein translation, requiring an efficient protein quality control system via regulation of autophagy and unfolded protein response mechanisms to ensure proteostasis [199,200]. It is not surprising that these biological processes are enriched when analysing GOs of selected myopathogenes (Fig. 4C and D). *KLHL40, HSPB8*, *DNAJB6, SQSTM1* and *VCP* are key players in the cellular chaperone/co-chaperone network that orchestrate the protein quality control system and unfolded protein response and are essential for proper stress response and protein turnover in mature muscle, where they support assembly of thin and think filaments [[201], [202], [203], [204]]. Inhibition of protein quality control system though, also inhibits muscle regeneration and contributes to satellite cell senescence [205], indicating potential roles in satellite cells.

Expression of *KLHL40* and *HSPB8* inversely correlate with that of *Pax7,* possibly through an indirect mechanism (Fig. 5), in keeping with their accumulation during exit from quiescence. Interestingly, expression of *LMOD3*, whose mutations lead to Nemaline myopathy 10 (NEM10; OMIM: 616165), also inversely correlates to Pax7 levels and is found reduced in mouse *Klhl40-*null muscles [203]. Mutations in HSPB8 are associated with distal hereditary motor neuropathy, Charcot-Marie-Tooth disease Type 2L (CMT2L; OMIM: 608673), and more recently with Neuronopathy, Distal Hereditary Motor, Type IIa (HMN2A; OMIM: 158590) [206]. Notably, HSPB8-myopathy appears to be due to a gain-of-function mutant *Hspb8* rather than its lack, as muscle function in homozygous knockout mice is marginally affected compared to knockin animals overexpressing a pathogenic version [207]. Mutations in *DNAJB6* are found in LGMDD1 (OMIM: 603511) where a proportion of patients suffer from facial weakness and respiratory disability, resembling clinical features of LGMD/Dystroglycanopathies. Therefore it is reasonable to infer an impairment of the muscle stem cell pool in these disorders, warranting examination of satellite cell status.

### 7.5 Skeletal muscle development, differentiation and contraction

The GO analysis also retrieved the biological processes skeletal muscle development, differentiation and contraction (Fig. 4C and D) from genes differentially regulated in satellite cells within 3 hours of activation. DYSFERLIN (encoded by *DYSF*) regulates calcium signalling across the plasmalemma. Our expression analysis reveals that *DYSF* is upregulated during satellite cell activation and its participation in myoblast fusion into myofibres is described [208]. Mutations in *DYSF* cause Dysferlinopathies, a group of muscular dystrophies composed of LGMDR2 (formerly LGMD2B; OMIM: 253601), Miyoshi Muscular Dystrophy 1 (MMD1; OMIM: 254130) and Myopathy, Distal, With Anterior Tibial Onset (DMAT; OMIM: 606768). All present with adult onset, progressive muscle weakness and myofibre necrosis with inflammatory infiltrates [209]. *DYSF* is also expressed in activated human satellite cells [210], and we found is downregulated upon Pax7 overexpression in mouse myoblasts (Fig. 5). Analysis of human and mouse DYSFERLIN-deficient muscles reveal unaltered numbers of quiescent satellite cells and *PAX7* expression [210,211]. However, patient biopsies show higher percentage of activated (MYOD-positive) satellite cells, possibly due to the hostile necrotic microenvironment [210], which is also reflected by increased serum CK in patients [209].

*HACD1* encodes the enzyme 3-Hydroxyacyl-CoA Dehydratase 1. HACD1 contributes to muscle growth through regulation of myocyte fusion and *HACD1* loss-of-function mutations are associated with a congenital myopathy [212]. While *Hadc1*-null mouse muscle has unaltered satellite cell numbers and repairs sufficiently, regenerated myofibres are smaller, suggesting inadequate satellite cell myogenesis [213].

Mutations in genes involved in the sarcomere seem unlikely to affect satellite cell function, but the recent description of a potential role for DYSTROPHIN highlights that they are worthy of consideration. Mutations in *ACTA1 encoding ACTIN α1* are associated with NEM3 (OMIM: 161800), while mutations in *TPM3 encoding TROPOMYOSIN3* associate with NEM1 (OMIM: 609284). Both disorders often involve respiratory distress, have increased satellite cells numbers and altered glucose metabolism [214], which together with progressive muscle atrophy, may suggest satellite cell involvement. Interestingly, both *ACTA1* and *TPM3* mutations are also associated with CFTD (OMIM: 255310), overlapping phenotypically with the satellite cell-opathy caused by SELENON-deficiency.

A similar increase in satellite cells was also observed in mice bearing mutation in the *TTN* gene encoding TITIN [215], although satellite cell numbers in human Titinopathies are unknown. Intriguingly, mutations in human *TTN* are associated with pathogenesis of Myopathy, Myofibrillar, 9, with Early Respiratory Failure (MFM9; OMIM: 603689), with the slowly progressive respiratory failure due to diaphragm weakness [216]. TITIN, along with FILAMIN C, are CALPAIN 3 targets and lack of FILAMIN C phenocopies LGMDR1, favouring an increase in satellite cell number in muscle bearing a *FLNC* pathogenic variant. *TTN* is also annotated within “Myofibre formation and maintenance”, which is specifically enriched in the upregulated geneset, consistent with the onset of satellite cell myogenesis. *TTN* is downregulated in response to Pax7 induction (Fig. 5), again suggesting an early role in satellite cell function (Fig. 4D and F).

Finally SH3 and Cysteine-Rich Domains *3* (STAC3) is a component of the excitation-contraction coupling machinery and is mutated in Myopathy, Congenital, Bailey-Bloch (MYPBB; OMIM: 255995), which exhibits distinct clinical overlap to CFZS, where satellite cells are dysfunctional [217]. Our analysis reveals *STAC3* accumulation in quiescent satellite cells, suggesting that its pathogenic variants may impair satellite cell function.

## 8 Next steps

Evaluation of the number of satellite cells and their function is required to further examine the role of satellite cells in some of these potential satellite cell-opathies. Such assessment in mouse models is well established [218,219] and can be performed in human muscle biopsies as a standard diagnostic approach (Fig. 3). Other established satellite cell markers are also available [220,221]. Primary and immortalised myoblasts, and Induced Pluripotent Stem Cells (iPSCs) also offer tools to examine satellite cell function [[222], [223], [224], [225]].

Another layer of complexity in classifying satellite cell-opathies comes from evidence of diversity in the muscle stem cell populations [226,227]. Satellite cell characteristics such as ability to self-renew, proliferation rate or extent of differentiation differ among muscles, or even between satellite cells from same muscle, further highlighting molecular heterogeneity [[228], [229], [230], [231], [232], [233]]. It has been suggested that individual satellite cells may transition across behavioural stages to maintain the dynamic equilibrium of the whole population [218,234,235]. Thus, different genetic variants found in muscular diseases may impinge more on specific satellite cell subpopulations, potentially influencing which muscles are affected in a particular disorder.

We chose the first 3 hours of satellite cell activation for our analysis (Fig. 4), but other points during satellite cell myogenic progression can be evaluated as data permits. This may identify satellite cell dysfunction resulting from mutation in other myopathogenes, as for example, *SELENON* was not differentially regulated during initial satellite cell activation, despite effects on satellite cell function. *CAPN3* was also not differentially regulated, further highlighting the ambiguous status of LGMD1R as a satellite cell-opathy.

Expression of the myopathogene *DNM2*, encoding DYNAMIN2, was also unchanged during this 3-hour time frame. Mutations in *DNM2* lead to a wide clinical spectrum including Centronuclear myopathy 1 (CMN1; OMIM: 160150), Lethal Congenital Contracture Syndrome 5 (LCCS5; OMIM: 615368); and Charcot-Marie-Tooth Disease, Dominant Intermediate B; (CMTDIB, OMIM: 606482). A knockin mouse model bearing a missense *DNM2* mutation recapitulates most CNM1 pathology, and displays both reduced satellite cell number and lack of regenerative potential [236,237]. Thus, alterations in *DNM2* may directly affect satellite cell status/function in CMN1, and likely LCCS5.

Finally, our analysis also highlights that Gene Ontologies referring to satellite cells (GOSC) should be revised and updated routinely to include recent literature and experimental observations. Surprisingly, the current GOSC annotation contains only 30 genes, omitting genes for which involvement in satellite cell biology is well established, such as *MYOD*, *POGLUT1* and *DAG1*. Improving GOSC annotation would contribute to a better definition, and clinical management, of satellite cell-opathies.

## 9 Summary

Here we analysed literature and used public transcriptomic datasets to examine satellite cell dysfunction across inherited muscular dystrophies and myopathies. We propose the term ‘satellite cell-opathy’ to collectively refer to muscle disorders where the mutation directly affects satellite cell function to contribute to pathology. This includes disorders such as MYOSCO, RSMD1, EMARDD, MYODRIF and CFZS. The pathogenic mutation may impair satellite cell specification, quiescence, activation, self-renewal and/or proliferation, which would affect the number of satellite cells and their function. Alternatively, the pathogenic mutation may only compromise satellite cell/myoblast myogenesis leading to defective differentiation/fusion (e.g. CFZS). We classify these conditions as ‘primary satellite cell-opathies’ (Table 1). However, further division may become possible as more disorders are identified.

There are common pathological features of a primary satellite cell-opathy. The most obvious is classification as congenital. General hypotonia is noted, with specific involvement of respiratory, trunk and facial muscles defining features (Fig. 2F, Table 1), although serum CK levels are usually within the normal range. Diaphragm for breathing, trunk muscles for posture/body support and facial/neck muscles for speech, mastication and facial expression undergo frequent use. If myofibres in these muscles are already smaller/fewer, such regular use may require chronic satellite cell activity. Mouse satellite cells in diaphragm and extra-ocular muscles self-renew at higher rates compared to those from limbs and have higher proliferative potential [238,239], so satellite cell dysfunction is likely to severely impact such muscles. Primary satellite cell-opathies are more unlikely to have cardiac involvement, unless the myopathogenes are involved in cardiac muscle development or expressed in cardiac non-muscle tissue.

Muscle disorders where the mutant myopathogene affects both satellite cell and myofibre function could be referred to as ‘secondary satellite cell-opathies’. Examples include congenital conditions such as MDCL and LAMA2-CMDs, disorders with juvenile onset such as FSHD, and adult onset for OPMD and DM1. In contrast, a Non-satellite cell-opathy neuromuscular disorder is where a myopathogene only affects muscle fibres and does not directly perturb satellite cell function and their regenerative response. However, in primary and secondary satellite cell-opathies, and non-satellite cell-opathy neuromuscular disorders, an increasing hostile microenvironment in muscle as disease progresses will also indirectly compromise satellite cell function and myofibre repair.

To further explore involvement of the 116 myopathogenes in satellite cell dysfunction, we created a multimodal process which interpolates 1) differential gene expression during mouse satellite cell activation [168], 2) regulation and binding of Pax7 in mouse [169], 3) regulation by human PAX7 [24] and 4) whether satellite cells are affected in the associated human disease and animal models. This identified several myopathogenes that could also directly affect satellite cells, with some of these associated disorders having perturbed satellite cell numbers/function, and prompting examination of satellite cell function in other conditions. Combined with clinical overlap of congenital onset and facial, trunk and diaphragm weakness in known primary satellite cell-opathies, our approach could help determine whether time-consuming histological evaluation of the stem cell pool is necessary for patient diagnosis. This could also advance assessment of genotype-phenotype correlation for several orphan diseases with similar clinical features. Finally, new myopathogenes, and genes from other disorders affecting skeletal muscle, can be subjected to our three way process to highlight potential effects on satellite cell function.

The definition of a primary satellite cell-opathy is a condition primarily caused by mutations in genes that lead to perturbed satellite cell function, while a secondary satellite cell-opathy involves mutations in genes that affect both satellite cells and muscle fibres (Table 1). Although such classification of satellite cell-opathies requires a multimodal approach, it represents a useful tool to improve diagnosis, delineate prognosis and accelerate ongoing development of tailored treatments for many inherited muscle conditions. This classification is also an important consideration when assessing the effectiveness of regenerative medicine therapies.

## Author statement

Conceptualisation: Massimo Ganassi and Peter S. Zammit. Data Curation and Analysis: Massimo Ganassi. Writing Original Draft: Massimo Ganassi and Peter S. Zammit. Review & Editing: Massimo Ganassi, Peter S. Zammit, Francesco Muntoni. Funding acquisition: Peter S. Zammit.

## Declaration of competing interest

Massimo Ganassi and Peter Zammit declare no conflicts of interest. Francesco Muntoni reports grants and personal fees from Sarepta, personal fees from Pfizer, personal fees from Dyne Therapeutics, grants from PTC, personal fees from Roche, outside the submitted work.
